# Training of young medical professionals: implementation of modern training concepts, taking into account the changed framework conditions for training—a pilot project for operational subjects

**DOI:** 10.1007/s00404-024-07447-9

**Published:** 2024-03-27

**Authors:** S. Findeklee, B. Haj Hamoud, K. Diedrich, R. M. Sima, E.-F. Solomayer, C. Spüntrup

**Affiliations:** 1 MVZ Göttingen, Kasseler Landstraße 25a, D-37081 Göttingen, Germany; 2 Department for Gynaecology, Saarland University Hospital, Obstetrics and Reproductive Medicine, Kirrberger Straße 100, Building 9, D-66421 Homburg, Germany; 3 University Hospital Schleswig–Holstein, Campus Lübeck, Frauenklinik, Ratzeburger Allee 160, D-23562 Lübeck, Germany; 4 Department for Obstetrics and Gynaecology, University Hospital “Carol Davila”, RO-020021 Bucaresti, Romania; 5 Saint John Hospital “Bucur” Maternity, RO-040294 Bucaresti, Romania; 6 Pelvic School Saarbrücken, Hohe Wacht 77, D-66119 Saarbrücken, Germany

**Keywords:** Laparoscopy simulator, Surgical simulation training, Evaluation, Student teaching

## Abstract

**Introduction:**

For years, generations of medical students have complained that practice-oriented learning is neglected in medical studies. Further training assistants also complain about limited opportunities to learn subject-specific practical activities.

**Material and techniques:**

We are presenting a pilot project at the University Women’s Hospital in Homburg, in which medical students complete an endoscopic hands-on course as part of the block internship gynaecology and obstetrics. During the course the students perform classic skills training and hand–eye coordination exercises and learn the first steps in endoscopic suturing (suture and rows of knots). The training concepts used can be implemented on simple boxing trainers and can therefore also be reproduced in clinics or in a private setting.

**Outcome:**

Altogether, 73 medical students did participate in the laparoscopy course. We were able to prove that the knotting time for a simple knot can be reduced from an average of 247 s to 40 s (80%) after completing our training programme. Based on the evaluation sheet that the students filled out after the course, we found a very-high acceptance for surgical simulation training within the student cohort.

**Discussion:**

Practical surgical exercises can complement the curriculum well and, as we can show with our work, are rated very positively by the students. For students in higher semesters, such practical courses can also provide an insight into the respective subject area and thus counteract the lack of skilled workers in surgical subjects. The practical year should not be the first contact with these practical courses, as at this timepoint a certain favoured subject has often already being chosen by the students.

## What dose this study add to the clinical work

**Table:** 

We suggest laparoscopic simulation training to be a suitable technique to enhance medical education. Whereas further studies are necessary to explore the long-term impacts of such training on clinical competency and patient care outcomes we think that laparoscopy trainers should become an integral part of the medical curriculum.	

## Introduction

In recent years, the framework conditions in operational training have changed in Germany. The traditional training system in form of learning surgical skills on the patient under the supervision of an experienced colleague can hardly be carried out in everyday routine. The specialization of surgical sub-areas, an intensification of work, scarce financial resources and also the change in social demands have meant that, e.g. in gynaecology, hysterectomy has a different status in specialist training than it did have 20 years ago. However, these changes are also accompanied by an increasing lack of young people in surgical subjects [1].

It is known that surgical interventions succumb learning curves. The number of repetitions being necessary to achieve a qualitatively good surgical level varies between 20 and 70 interventions, depending on the complexity of the intervention. Laparoscopic surgery in particular is known for its rather flat learning curve [2, 3].

This raises the question of how practical procedures, such as laparoscopic surgery, can be learned at a time in which the resources for training in everyday clinical practice are reaching their limit. Besides, for ethical reasons, it has also be discussed, whether, surgical skills training still can be carried out on patients instead of training simulators.

Comparable to the pilot training, that has been established for decades, there are now numerous simulators for endoscopic operations with which good success concerning learning curves has been demonstrated [4–6]. This training on the simulator is usually offered subject-specific or partially operation-specific as part of chargeable courses [7]. Course fees and travel expenses are usually borne by the course participants themselves—in contrast to pilot training, for example. However, in medicine—in contrast to pilot training—the possibility of using surgical simulators at every clinic nationwide is very limited [8].

The aim of this original article is to present the concept and the evaluation results of a laparoscopy course for future medical doctors to motivate other medical faculties and clinics in Germany to consider simulated training as a basis for surgical training. The concept presented by us allows an ethically responsible training with an acceptable expenditure of time. With regard to the financial outlay, the system presented can be more economical for the clinics than the traditional training system, since the learning curve plateaus can be reached without costly operating times and the complication rates may also be positively influenced.

In our opinion it makes sense to place the first contact with such simulators in a late phase of the medical school, since the relevant professional fields can be explored in a vivid way and, ideally, the lack of skilled workers in surgical subjects can be counteracted.

## Material and techniques

Medical students who took part in the so-called block internship (9th semester) were offered the opportunity of participating in laparoscopic simulation training over a period of 2 hours as part of the gynaecology internship. Since a maximum of ten students is usually assigned to each block, five workstations were made available.

A working place included the following working material:

- a classic boxing trainer with accessories (the accessories did consist of a bead insertion tool, a pipe cleaner tool, a suture pad, an endoscopic gripper, an integrated light source and a tablet/smartphone holder);
- an endoscopic needle holder for practice purposes (can be purchased separately);
- a tablet/smartphone (as a substitute for the camera, provided by the students or by us).

**Fig. 1:**
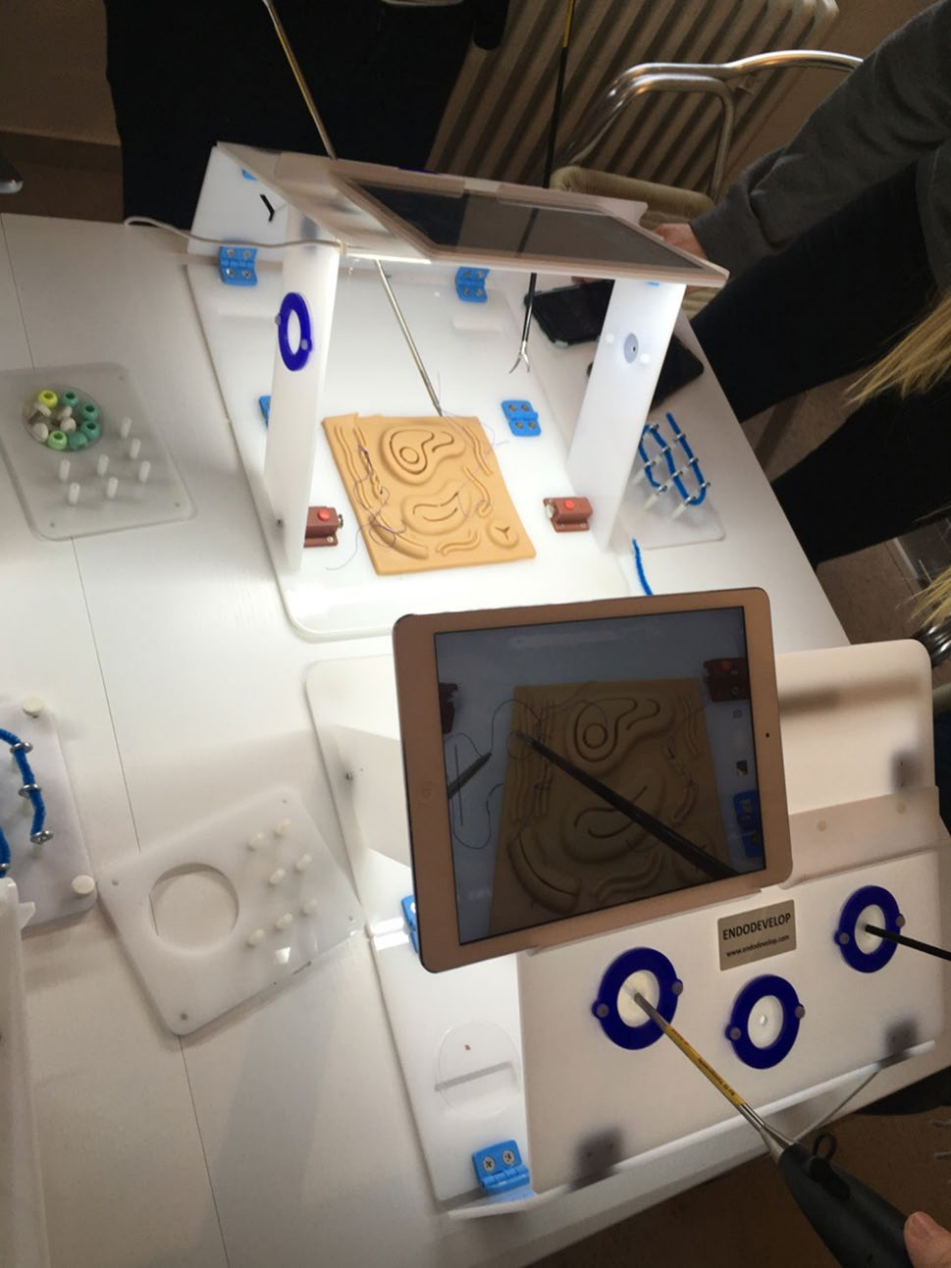
Practical laparoscopy exercises performed on the simulator by the students

We used an approx. 20 square meter room with a 1 m × 2.40 m table. Alternatively (depending on the number of simulators), several small tables would also suffice (see Fig. 1).

The students each practiced alone at a training station. If there were more than five participants, the students took turns at the free stations after completing the exercise.

The curriculum with short explanation of the instruments did include the following steps:-short explanation of the instruments

- short explanation of the instruments

### Exercises on the bead tool (10–15 min)

Aim: Hand–eye coordination training and transition from one-handed to two-handed training.

1. This exercise is done with one hand. Threading thick beads with a hole diameter of 6 mm onto appropriate rods. The students are instructed to grasp the bead with the gripping instrument and then spread the instrument. It can then be placed on the staff in a controlled manner.
2. From now on, you will operate with two hands. In the second hand, there is a needle holder with which the threaded bead can be undermined and pushed up so far that the gripper of the other hand can be used to grip the bead from above. This is then put back into the collection bowl.
3. Now, go through the bead opening with the needle holder. With the gripper of the other hand, the pearl is pushed up and fixed. Then proceed with the other beads in the same way until all beads are threaded.

### Exercise on the pipe cleaner tool (approximately 10–15 min)

This classic hand–eye coordination exercise involves sliding a pipe cleaner through an array of grommets. The main difficulty is that the work is three-dimensional, but is only transmitted two-dimensionally on the monitor. In addition, the pipe cleaner must be pulled through sufficiently from the start so that all eyelets can be grasped. This exercise also teaches you how to dose the lever and pulling forces.

### Suture and knot exercises (approximately 70–90 min)

First, the students are shown how to clamp the needle correctly. This is followed by a demonstration of the correct stitch technique and a possibility of knotting.

The students should then first carry out the following exercises with Vicryl 2–0 threads with a large needle on a straight strand of the suture pad:

1. clamping the needle;
2. puncture;
3. double knot and single reverse knot;
4. continuous seam with three stitches, whereby the thread is not completely pulled through at the last stitch;
  At this point, the technique of tying the final knot in a running suture is demonstrated to the students. They do this afterwards;
5. closing knot;
6. further rows of seams at other points on the seam pad with smaller needle sizes, thinner threads and PDS threads and additional knots.

Additional modules are available for colleagues who are already surgically advanced. These modules can simulate more complex interventions such as descenus operations, cyst enucleations or appendectomies and can be operated on accordingly.

### Evaluation of the simulation training by the students

#### Evaluation form

We used a standardized-evaluation form for the course evaluation by the participants. This comprised 15 statements. To assess these statements, one could choose between the five categories “applies”, “rather applies”, “neutral”, “rather does not apply” and “does not apply” (with a cross) (see Table 1). The evaluation forms were completed and submitted anonymously.

**Table 1:** Evaluation results of the laparoscopy course by the students (*n* = 73)

Category	1 (Applies)	2 (Rather not applicable)	3 (Partly applies)	4 (Rather not applicable)	5 (Not applicable)
Using the models in the laparoscopy course has improved my manual skills	66 (90.4%)	6 (8.2%)	1 (1.4%)	0	0
Using the models has improved my ability to utilize laparoscopic instruments	65 (89%)	7 (9.6%)	1(1.4%)	0	0
The application of the laparoscopic models helped me to become aware of the algorithm of action in a minimally invasive gynaecological intervention	51 (69.9%)	15 (20.5%)	6 (8.2%)	0	1 (1.4%)
I did benefit greatly from the practical laparoscopic exercises on the models	64 (87.7%)	9 (12.3%)	0	0	0
As part of my overall experience, the hands-on laparoscopic exercises on the models have improved the quality of my medical studies	62 (84.9%)	9 (12.3%)	2 (2.7%)	0	0
I wish to do more practical laparoscopic exercises with laparoscopic models in the Practical year	70 (95.9%)	3 (4.1%)	0	0	0
I wish to do more hands-on training with laparoscopy trainers in other medical subjects	70 (95.9%)	2 (2.7%)	1 (1.4%)	0	0
The laparoscopic models have improved my understanding of the subject gynaecology and obstetrics	32 (43.8%)	19 (26%)	18 (24.7%)	2 (2.7%)	2 (2.7%)
The practical laparoscopic exercises have improved my understanding and competence of a surgical procedure	49 (67.1%)	23 (31.5%)	1 (1.4%)	0	0
I have received sufficient technical knowledge about the processes in the operating room	32 (43.8%)	22 (30.1%)	16 (21.9%)	3 (4.1%)	0
I have gained enough self-confidence to work in the operating room	26 (35.6%)	17 (23.3%)	22 (30.1%)	5 (6.8%)	3(4.1%)
I was motivated during the practical exercises on the laparoscopic models within the semester	66 (90.4%)	7 (9.6%)	0	0	0
The practical laparoscopic exercises did motivate me to work in the field of gynaecology and obstetrics in the future	23 (31.5%)	20 (27.4%)	8 (11%)	12 (16.4%)	10 (13.7%)
I enjoyed the practical exercises with the models	67 (91.8%)	6 (8.2%)	0	0	0
I learned to work in a team	32 (43.8%)	16 (21.9%)	20 (27.4%)	1 (1.4%)	4 (5.5%)
The practical exercises with the laparoscopic models are relevant for my future medical profession	47 (64.4%)	15 (20.5%)	6 (8.2%)	5 (6.8%)	0
The practical exercises with the laparoscopic models would have relevance for my later medical profession if I would work in the field of gynaecology and obstetrics	52 (71.2%)	13 (17.8%)	6 (8.2%)	1 (1.4%)	1 (1.4%)
I would recommend the event	68 (93.2%)	5 (6.8%)	0	0	0

## Outcome

### Active part of the course

The skills exercises and the hand–eye coordination exercises could be carried out by the students in adequate time. Students who have problems with the first exercises can compensate this within the next exercises. After the 2-h course, over 90% of the students were able to insert a needle endoscopically and to tie knots. The first knots were not always tightened correctly (“air knots”), although this improved with the number of knots made and it was also critically realized by the students. The more the continuous seams are performed, the neater the seam rows will be. More than 90% of the students managed to sew three rows of stitches with at least six to eight knots within the given period of time. The seam and knotting times are progressively shorter (time saving between the first versus last knot in an interim evaluation in the current course was 75%, i.e. approximately 4 min).

### Examination of the evaluation sheets

A total of 78 medical students took part in the laparoscopy course during the block internship. Of the 78 students, 73 (93.6%) completed the course evaluation form completely. The evaluation form was not filled out completely for three times. Two single answers were missing.

The conclusion of the students during the evaluation of the course was consistently positive: 95.9% (70/73) wanted to receive more practical exercises of this type in other subjects and in the practical year. Sixty-eight out of 73 participants (93.2%) would unreservedly recommend the course with the laparoscopy simulator. Sixty-seven out of 73 students (91.8%) summed up that they thoroughly enjoyed the course. Sixty-six of 73 participants (90.4%) were fully motivated. Sixty-five medical students (89%) felt that using the models had unreservedly improved their ability to use laparoscopic instruments. Sixty-four students (87.7%) did possess the opinion that they had benefited greatly from the practical laparoscopic exercises on the models. Sixty-two participants (84.9%) saw an improvement in the quality of medical studies as a result of the course with the laparoscopy simulator. Forty-nine (67.1%) unreservedly agreed that the course improved understanding and competence of a surgical procedure. Forty-seven course participants (64.4%) had the opinion that the course was relevant for their later medical profession, even more in the case of a later gynaecological occupation (52 or 71.2%). At least 32 students (43.8%) found unreservedly that the laparoscopy simulation course improved their understanding of the subject of gynaecology and obstetrics, that the course gave them sufficient technical knowledge of the processes in the operating room and that they learned to to work as a team.

The lowest level of agreement was observed for the statements that the participants in the course had gained enough self-confidence to work in the operating room (26 times full agreement or 35.6%) and that the course did motivate the participants to commence a work in the subject gynaecology and obstetrics (23 students or 31.5% fully agreed).

The evaluation results of the laparoscopy course by the medical students are summarized in Table 1.

## Discussion

The pilot project presented shows that laparoscopic training on the simulator with a structured training programme reveals good results in terms of laparoscopic basic skills (skills, hand–eye coordination, suture and knot techniques) even in a short time and is used by trainee colleagues as an alternative to training on the patient.

The use of simple boxing trainers in combination with modern tablet cameras enables the affordable provision of a sufficient number of simulator units so that a larger number of trainees can be trained simultaneously. This allows economic teaching—both in terms of time and money. Using tablet cameras as optics, modern boxing trainers allow self-sufficient practice without the inevitable need for expensive endoscopy optics and surgical assistance, but still allow this option as an alternative. A wide availability of inexpensive simulators with the possibility of structured training up to junior status could favour the use as a recognized training tool.

Numerous studies have shown that basic laparoscopic skills on simulators provide an impressive improvement in the learning curve. In a teaching project on our training concept, we were able to prove that the knotting time for a simple endoscopic knot decreased from an average of 247 s (range 45–1290 s) to 40 s (range 7–280 s) after our 2-h training session, i.e. by more than 80 percent [9] Calculating with costs of 40–50 € for an average minute in the operation room there would result the potential of cost savings of about 155 € per endoscopic knot if the operation is performed by a junior surgeon [10].

From a behavioural point of view, repetition is an important factor in implementing what has been learned. It could be shown that a first learning plateau is already reached after 10 knots [9]. Ideally, there should be at least one night between two training sessions. Unfortunately, this can often not be set up in everyday clinical practice for teaching. Nevertheless, it could be proven that the trainees start at a higher level and improve their learning curves more quickly than it was initially the case, even with longer time intervals when resuming the simulator training [11].

Our students have the opportunity to continue to use the existing university simulators so that the level of learning can be gradually increased. Training videos of the taught content can be viewed free of charge on the internet by both, us and other surgeons, so that not only repetition but also further development of skills is possible on the basis of the teaching content we provide. Further development from novice status to junior status is also possible at low cost—similar to pilot training—with modern boxing trainers that contain the appropriate devices for more complex, subject-specific operations.

Academic teaching hospitals and university clinics often offer the opportunity of taking on surgical assistance to medical students during their practical year. Although there is no exact data for equivalent value calculations, the degree of improvement in endoscopic knotting times after brief training on the simulator implies that training on the simulator also has a positive effect on surgical assistants [12]. At the latest, when the training assistants find themselves in the role of the surgeon under the supervision of an experienced person, the more than 80% improvement in knotting times will also pay off financially for the hospital authorities.

Last but not least, the ethical aspect should also be taken into account, since it is obvious that the surgical complication rate of laparoscopic operations could be reduced by primary training on the simulator and not on the patient [13, 14]. In the USA, therefore, primary training on simulators is now required in numerous clinics before procedures can be carried out on humans. There are numerous simulation centres for this, which then have interdisciplinary simulators at their disposal. University centres in particular often have their own or affiliated simulation centres. The availability of training opportunities without major effort is known to increase their use.

Conversely, it is hardly to be expected that trainees will use simulators and equipment if these do not technically work. The construction involves a great deal of effort or the optics only deliver good images with great effort.

The laparoscopic simulators currently available on the market enable differentiated training goals. Biological simulators (animal carcasses, body donors) do not offer pathologies that can be planned, but are extremely realistic with regard to the operational structures. Nevertheless, simulators still have serious weaknesses regarding haptic feedback quality and some also possess reserves concerning image quality. A future perspective could be the implementation of the new metaverse technologies virtual reality (VR) and augmented reality (AR) having a grand potential to enhance training experiences into surgical simulation programmes. Virtual reality trainers (VRT) can simulate complex operations with any pathology comparable to a video box. They allow self-sufficient practice without the need for camera work. A partially not yet ideal haptic impression and the close connection to the programmed surgical procedures limit a comprehensive application in addition to the very-high acquisition costs. Clinics that own Da Vinci© systems have the option of using the integrated training tools. However, these systems are not usually utilized for the initial steps in surgeon training.

Boxing trainers are a good option for skills training, hand–eye coordination as well as suturing and knotting techniques. Modern boxing trainers have additional options such as the use of alternative optics or the implementation of partial operational steps and complete operations. The combination with animal carcasses (e.g. upper belly package of the pig) is also possible. We have favoured the use of modern boxing trainers for our pilot project, as they can also be used by surgical novices with little effort and provide good training results without harming a living being.

A relevant question in the discussion about the training of medical colleagues in Germany is often who ultimately has to bear the costs for travel, accommodation and course fees. In Germany, the costs for topic-specific medical training beyond the course of study, e.g. in the context of courses or symposiums, are usually borne by the colleagues themselves and, if at all, are usually only financially supported to a lesser extent by the clinics [15].

In the case of pilot training, the costs of the training are largely borne by the employer. Large airlines in particular have their own simulators. One possibility for cost control for the simulated training, based on the USA, would be the purchase of simulators by the clinics themselves. The clinics are free to choose which simulators they ultimately choose. With training concepts like ours, the basic principles can be implemented in a short time. The availability of the simulators in the clinics would increase their use as a continuous training tool. There would be no travel or accommodation costs for the clinics or the young surgeons. The medical faculty day found that practical training content still has insufficient value in the course of studies. Practical OSCE (Objective Structured Clinical Evaluation) exams were stipulated as a mandatory part of teaching in the 2020 master plan for medical studies [16]. Practical surgical exercises would complement the curriculum well and, as we can show with our work, are rated very positively by the students. For students in higher semesters, such practical courses can also provide an insight into the respective subject area and thus counteract the lack of skilled workers in surgical subjects. The practical year should not be the first contact with these practical courses, as at this timepoint a certain favoured subject has often already being chosen by the students [17].

Our study has several serious limitations which have to be critically discussed. First of all, we did include a limited number of participants (73 students undergoing the complete block internship for gynaecology and obstetrics). Whereas self-reported outcome measures provide valuable insights into the students perceptions and satisfaction, they also have the potential of bias, since sometimes there is the tendency to give answers which are expected by the investigators. Integrating objective performance metrics could offer a more comprehensive evaluation of the training’s effectiveness like OSCE assessments. This could include standardized assessments of surgical skills and techniques by independent evaluators, as well as tracking clinical outcomes when these students begin to practice.

Furthermore, we are planning to perform a future study including a control group of students, who undergo traditional training without the aid of simulation. This comparison would allow for a more definitive assessment of the simulation training’s effectiveness over conventional methods. Besides, implementing a longitudinal study design to follow participants over time would help in assessing the long-term retention of skills gained through simulation training. It would also allow for the evaluation of how these skills translate into clinical practice and affect patient care outcomes. In addition, assessing how simulation training can be best integrated with clinical rotations to maximize learning outcomes could be beneficial. This includes determining optimal timing and sequencing for simulation training to complement traditional clinical experiences. Over and beyond, introducing structured feedback mechanisms for students to share their experiences and suggestions for improving the simulation training programme could foster continuous improvement. This feedback could inform updates to simulation scenarios, equipment, and instructional methods.

Finally, addressing scalability and accessibility issues related to simulation training is crucial. This includes exploring strategies to make simulation training more accessible to institutions with limited resources (e.g. non-academic hospitals) and identifying best practices for scaling these programmes effectively. This also includes research into technologies that can be applied globally, especially in developing countries, without major financial outlay.

In conclusion, our study offers insights into the potential of laparoscopy simulation training to enhance medical education. While the results are promising, there is a clear need for further research to explore the long-term impacts of such training on clinical competency and patient care outcomes.

## Data Availability

The data are available from the authors.
